# Photobiomodulation promotes spinal cord injury repair by inhibiting macrophage polarization through lncRNA TUG1-miR-1192/TLR3 axis

**DOI:** 10.1186/s11658-023-00417-0

**Published:** 2023-01-19

**Authors:** Cheng Ju, Yangguang Ma, Xiaoshuang Zuo, Xuankang Wang, Zhiwen Song, Zhihao Zhang, Zhijie Zhu, Xin Li, Zhuowen Liang, Tan Ding, Xueyu Hu, Zhe Wang

**Affiliations:** Department of Orthopedics, Xijing Hospital, Fourth Military Medical University, Changle West Road No. 127, Xi’an, 710032 Shaanxi China

**Keywords:** Spinal cord injury, Bone-marrow-derived macrophages, Photobiomodulation, Transcriptome sequencing, Inflammation, Long noncoding RNA TUG1

## Abstract

**Background:**

Secondary spinal cord injury (SCI) often causes the aggravation of inflammatory reaction and nerve injury, which affects the recovery of motor function. Bone-marrow-derived macrophages (BMDMs) were recruited to the injured area after SCI, and the M1 polarization is the key process for inducing inflammatory response and neuronal apoptosis. We previously showed that photobiomodulation (PBM) can inhibit the polarization of M1 phenotype of BMDMs and reduce inflammation, but the underlying mechanisms are unclear. The purpose of this study is to explore the potential target and mechanism of PBM in treating SCI.

**Methods:**

Transcriptome sequencing and bioinformatics analysis showed that long noncoding RNA taurine upregulated gene 1 (lncRNA TUG1) was a potential target of PBM. The expression and specific mechanism of lncRNA TUG1 were detected by qPCR, immunofluorescence, flow cytometry, western blotting, fluorescence in situ hybridization, and luciferase assay. The Basso mouse scale (BMS) and gait analysis were used to evaluate the recovery of motor function in mice.

**Results:**

Results showed that lncRNA TUG1 may be a potential target of PBM, regulating the polarization of BMDMs, inflammatory response, and the axial growth of DRG. Mechanistically, TUG1 competed with TLR3 for binding to miR-1192 and attenuated the inhibitory effect of miR-1192 on TLR3. This effect protected TLR3 from degradation, enabling the high expression of TLR3, which promoted the activation of downstream NF-κB signal and the release of inflammatory cytokines. In vivo, PBM treatment could reduce the expression of TUG1, TLR3, and inflammatory cytokines and promoted nerve survival and motor function recovery in SCI mice.

**Conclusions:**

Our study clarified that the lncRNA TUG1/miR-1192/TLR3 axis is an important pathway for PBM to inhibit M1 macrophage polarization and inflammation, which provides theoretical support for its clinical application in patients with SCI.

**Supplementary Information:**

The online version contains supplementary material available at 10.1186/s11658-023-00417-0.

## Background

Spinal cord injury (SCI) is mostly caused by external factors, which can cause motor, sensory, and autonomic nerve injury [1, 2]. Due to the lack of effective treatment and poor prognosis, SCI causes a heavy emotional and economic burden to patients, family, and society [3, 4]. Primary injury after SCI is difficult to prevent, but the subsequent secondary injury can lead to a series of cascade reactions that can continuously aggravate the injury [5, 6]. Among them, secondary inflammatory injury is an important factor hindering SCI repair. After secondary injury, the inflammatory response is activated, and bone-marrow-derived macrophages (BMDMs) are recruited to the injured area [7]. BMDMs were polarized to M1 and M2 phenotypes in the injured area. The M2 phenotype secretes high levels of IL-10, TGF-β, Arg1, and neurotrophic factors, which inhibit inflammatory responses and promote nerve regeneration [8]. Instead, the polarization M1 macrophages can aggravate the inflammatory response, increase neuronal apoptosis, and further worsen the progression of SCI [9]. By contrast, M1 polarization occupies the main direction in the damaged state. Therefore, effectively inhibiting the inflammatory response induced by M1 macrophages is extremely important for the treatment of SCI.

Photobiomodulation (PBM) therapy, is a safe, noninvasive, and simple physical therapy that can trigger beneficial biological reactions in cells and tissues by directly applying low-energy lasers to specific areas [10]. Several studies have shown that PBM has the ability to regulate inflammation, wound healing, nerve regeneration, and osteogenic differentiation [11–13]. In our previous studies, we verified the safety and stability of PBM in the treatment of SCI in piglet model, and found that PBM could inhibit the M1 polarization of BMDMs and alleviated inflammation [14, 15]. However, the potential targets and mechanisms of PBM in the treatment of SCI remain unclear.

Only 2% of the transcripts in the human genome encode proteins, and at least 75% of the transcripts are noncoding RNA (nc-RNA). A considerable number of transcripts are composed of more than 200 bp, which are called long noncoding RNA (lncRNA) [16]. Although lncRNA cannot encode proteins, it still plays an important role in the occurrence and development of diseases [17]. The biological function of lncRNA can be performed from the promoter region, exon, antisense sequence, enhancer sequence, untranslated region (UTR), intergene, and intragene regions of the genome [18]. lncRNA is involved in epigenetic and transcriptional gene regulation, including histone modification, DNA methylation, and chromatin remodeling [19]. In addition, lncRNA can regulate gene expression both transcriptionally and posttranscriptionally [20]. For example, it can interact with miRNA, affect proteins in the cytoplasm, and regulate RNA metabolism [21]. It plays an important role in regulating cell cycle, proliferation, metastasis, immunity, and differentiation [22]. However, whether PBM can perform its biological function through lncRNA has not been reported.

In this study, to explore the potential mechanism by which PBM regulates the polarization of BMDMs, we identified a critical ceRNA network and explored its function. The characteristics of this network provide a theoretical basis for PBM treatment of SCI.

## Materials and methods

### Animals

All C57BL/6 male mice (6–8 weeks) were purchased from the Animal Experimental Center of the Fourth Military Medical University, and all mice were raised in a standard environment as described previously [15]. The whole animal experiment scheme has been approved by the Animal Ethics Committee of the Fourth Military Medical University (approval no. IACUC-20210358).

### Extraction and culture of BMDMs

C57BL/6 mice were killed and sterilized in 70% ethanol for 15 min. The intact femur and tibia of the hindlimb of mice were removed with sterile instruments. The bone marrow cavity was washed repeatedly with precooled phosphate-buffered saline (PBS), and the completely mixed suspension was filtered and collected in a 15 mL centrifuge tube. Red blood cell lysate was added in the ratio of 1:3 to lyse for 10 min. After centrifugation (300*g* × 5 min), the supernatant was discarded, and the cells were gently resuspended and cultured in a modified Dulbecco medium containing 10% fetal bovine serum and 10 ng/mL macrophage colony-stimulating factor (MCSF). All cells were cultured in 37 °C incubator containing 5% CO_2_ for 7 days until maturation. M1 polarization of BMDMs was induced by LPS (100 ng/mL, Sigma-Aldrich, USA) + INF-γ (20 ng/mL, PeproTech, USA).

### Immunofluorescence

The cells were fixed at room temperature with 4% paraformaldehyde for 20 min. The frozen sections of spinal cord tissue or cells were washed with PBS three times, and then incubated with 0.3% Triton X-100 for 30 min. Bovine serum albumin (BSA) was used to block for 30 min and incubated overnight with primary antibodies at 4 °C. The primary antibodies used include anti-F4/80 (cat. no. ab6640, Abcam,1:300), anti-iNOS (cat. no. 13120, Cell Signaling Technology, 1:300), anti-MAP2 (cat. no. 8707, Cell Signaling Technology, 1:300), anti-β-III-tubulin (cat. no. ab78078, Abcam, 1:300), and anti-NeuN (cat. no. ab177487, Abcam,1:300). The next day, the second antibodies was incubated at room temperature for 1 h and the nucleus was stained with DAPI. Finally, the fluorescence image was obtained under a fluorescence microscope (BX51, Olympus).

### Flow cytometry

The cells were collected after 48 h of treatment and resuspended with PBS. Under dark conditions, F4/80 antibody (APC-F4/80, 1:50, eBioscience, cat. no. 17-4801-82) was added to M0 macrophages, F4/80 and CD86 antibody (PE-CD86, 1:200, BioLegend, cat. no. 105014) were added to M1 macrophages, and then incubated for 30 min at 4 °C. Identification and detection of macrophages by flow cytometry (Beckman Coulter, CA, USA).

### Transcriptome sequencing analysis

BMDMs were harvested after treatment, and total RNA was extracted with TRIzol reagent. Our sequencing was divided into three groups, including M0 group, M1 group, and M1 + PBM group (*n* = 3 per group). Genergy Biotechnology Co. Ltd (Shanghai, China) performed enrichment, fragmentation, reverse transcription, library construction, sequencing, and data analysis. Fastq-formatted raw data were processed and analyzed. The number of transcripts in each sample was calculated according to fragments per kilobase of transcript per million fragments mapped (FPKM). For each sample, FPKM values were calculated using Cuffnorm software, and log_2_ transformations were applied. The differential gene expression between different samples was calculated using DESeq2 software. The threshold of differentially expressed transcripts was determined to be *P* < 0.05 and multiple change ≥ 1. The KEGG database was used to analyze the signal pathway enrichment of differentially expressed transcripts. When *P* < 0.05 and at least two genes are involved, the pathway of significant enrichment can be determined. The STRING database (https://string-db.org/) is used to construct PPI networks for DEmRNAs, download interaction data, and analyze hub genes with Cytoscape software. miRNAs-target lncRNA TUG1 and miRNAs-target mRNAs were predicted by starBase database (https://starbase.sysu.edu.cn/). Comprehensive ceRNA score and expression value prediction results were used to screen ceRNA. The main data were uploaded to the NCBI database (login number PRJNA780778).

### Cell transfection

The knockdown and overexpression adenovirus of TUG1 was synthesized by Hanbio Biotechnology (Shanghai, China), and the optimal multiplicity of infection (MOI) was selected for transfection. TLR3 siRNA, miRNA-1192 mimics and inhibitors were purchased from GenePharma Co. Ltd (Shanghai, China). Lipofectamine 2000 was used as transfection reagent. Poly(I:C) (Sigma-Aldrich, MO, USA) was used to stimulate the expression of TLR3. All reagents are transfected according to the manufacturer’s instructions.

### Cytoplasmic and nuclear fractionation

According to the manufacturer’s instructions, Minute Cytoplasmic and Nuclear Extraction Kits (Invent Biotechnologies, Berkshire, Plymouth, USA) were used to separate the cytoplasm and nucleus of macrophages. The isolated extract was dissolved with TRIzol reagent to extract RNA, and the subcellular localization of TUG1 was detected by RT-PCR. GAPDH and U6 were used as cytoplasmic and nuclear controls, respectively.

### SCI model

The SCI model is constructed as previously described [23]. Mice were anesthetized by intraperitoneal injection of 0.6% sodium pentobarbital, and their back hair was removed. The mice were fixed on a sterile operating table and disinfected with 75% medical alcohol. T9 was taken as the center to make a longitudinal incision. The skin and subcutaneous tissue was cut in turn, the spinous process and lamina of T8–T10 were exposed, and T9 lamina under microscope was removed to completely expose the spinal cord. The modified forceps was used to clamp spinal cord tissue (vertical direction) for 30 s to cause SCI, then hemostasis and suture. After SCI, manually squeeze the bladder to urinate every day, and observe the vital signs of mice. The control group only underwent laminectomy.

### Photobiomodulation therapy

SCI mice were randomly divided into PBM treatment group and injury group. The mice were anesthetized by intraperitoneal injection of 0.6% sodium pentobarbital and placed in a dark cage (temperature 25 °C). An 808 nm laser device (MW-GX-808/1000 mW, near-infrared spectrum) made by Changchun Leishi Optoelectronic Technology Co., Ltd and its supporting medical diffusion optical system were used for PBM treatment in mice. The safety and irradiation parameters of optical fibers have been verified in piglets [14, 24]. The medical highly transparent silica coating on the surface of optical fiber ensures its flexibility and biocompatibility without affecting its optical properties. The optical fiber is cylindrical with a diameter of 600 μm. Use a calibrated optical sensor to confirm that the output power of the optical fiber is consistent with the set power. We irradiated the spinal cord injury area of mice for 50 min every day (50 mW/cm^2^). At the cellular level, the cells are placed on an ultraclean worktable and exposed to 808 nm low-level laser irradiation every 12 h. The specific parameters are described in Additional file 2.

### Quantitative real-time PCR

After the cells were treated (the spinal cord tissue was removed and ground), the total RNA was extracted with TRIzol reagent according to the manufacturer’s instructions. cDNA was obtained using Evo M-MLV RT premix reagent (AG11706, Accurate Biotechnology, China). The reaction conditions are 37 °C, 15 min, 85 °C, 5 s, and 4 °C 10 min. SYBR Green is used for quantitative real-time PCR (qPCR). CFX (Invitrogen, Waltham, Ma, USA) and CFX connect real-time PCR system (BioRad, Hercules, CA, USA) were used for 15 s at 95 °C, followed by 40 cycles at 95 °C for 5 s and 34 s at 60 °C. Use the 2^−ΔΔCT^ method to analyze the data. miRNA-1192 primers and the internal reference U6 were synthesized by General Biology Co., Ltd. All primers are listed in Additional file 3.

### Western blotting analysis

The cells were washed with PBS and then lysed on ice with RIPA buffer containing phosphatase inhibitor (the spinal cord tissue in the injured area was fully ground after adding RIPA buffer containing phosphatase inhibitor). All proteins were harvested and transferred to a 1.5 mL centrifuge tube after 20 min of cleavage, and the precipitate was discarded after 20 min of centrifugation (4 °C × 12,000*g*). BCA protein analysis kit is used to detect protein concentration (Thermo Scientific, 23227). The total protein extract was separated by SDS-PAGE and transferred to nitrocellulose membrane (P-N66485, Pall, America). The nitrocellulose membrane was sealed at room temperature for 1 h in 5% skim milk and then incubated overnight with primary antibody at 4 °C. Primary antibodies: iNOS (cat. no. 13120, Cell Signaling Technology, 1:1000), TLR3 (cat. no. ab62566, Abcam, 1:1000), p-NF-κB (cat. no. 3033, Cell Signaling Technology, 1:1000), and β-actin (cat. no. 66009-1-IG, Proteintech, 1:3000). The next day, the secondary antibody was incubated at room temperature for 1 h, and the Amersham Imager 600 (General Electric) was used for imaging after adding ultrasensitive luminescent solution.

### Neuronal culture and treatments

The dorsal root ganglion (DRG) was extracted from Sprague Dawley rat neonates (P1–P3) using the method we previously reported [25]. The DRG was completely cut, digested with trypsin digestion solution (0.125%) and type IV collagenase solution (0.1%) for 30 min, and then supplemented with 20% FBS DMEM–F12 to stop digestion. The cells were centrifuged at 1000 rpm for 5 min and then suspended in a medium with addition of B27 and 1% penicillin/streptomycin and cultured in a 12-well plate.

To explore the effect of BMDMs on DRG toxicity, we collected the culture supernatant of BMDMs, including M0 macrophage-conditioned medium (MCM), M1-MCM, shTUG1-MCM, shTUG1 + PBM-MCM, OE-TUG1-MCM, and OE-TUG1 + PBM-MCM. The conditioned medium was filtered with 0.22 mm membrane to remove the cell residue. Half of the medium of DRG was replaced by MCM. After neurons were cultured in mixed medium for 24 h, immunofluorescence was used to evaluate the effect of MCM on neuronal axon growth.

### Luciferase assay

The wild-type (WT) or mutant-type (MUT) TUG1 3′-UTRs and TLR3 3′-UTRs were cloned into pmir-GLO plasmids. Compared with negative control (NC) mimics, miR-1192 mimics and TUG1 3′-UTRs or TLR3 3′-UTRs were co-transfected into 293T cells (National Collection of Authenticated Cell Culture, China, cat. no. GNHu17) with Lipofectamine 2000 (Invitrogen, USA). The Luciferase Reporter Assay System was used to detect luciferase activity.

### Fluorescence in situ hybridization

The spinal cord tissues of mice from different days were collected for frozen sections, and an lncRNA FISH kit (GenePharma, Shanghai, China) was used for RNA fluorescence in situ hybridization. According to the instructions, the frozen sections were rehydrated, digested with protease K, denatured, hybridized with TUG1 nucleotide probe, stained with DAPI, and then observed under a fluorescence microscope.

### Functional assessment

The Basso mouse scale (BMS) was used to evaluate the recovery of motor function in mice at 1, 3, 7, 14, and 28 days after injury. The footprint was used to evaluate the step length recovery of mice by gait analysis after 28 days of PBM treatment. Two researchers who did not participate in the experiment performed functional assessment.

### Statistical analysis

All the experiments were repeated at least three times independently. The data statistics of this study were processed by GraphPad Prism software (8.3.0 version). Student’s *t*-test was used for comparison between two groups. One-way analysis of variance (ANOVA) with least significance difference post hoc analysis was used for comparison of three groups or more. The measured data are presented as mean ± standard deviation (SD). ImageJ software was used to perform optical density statistics, axon length measurement, and positive cell count. *P* < 0.05 was considered to be statistically significant.

## Results

### Identification and induction of macrophages

**Fig. 1:**
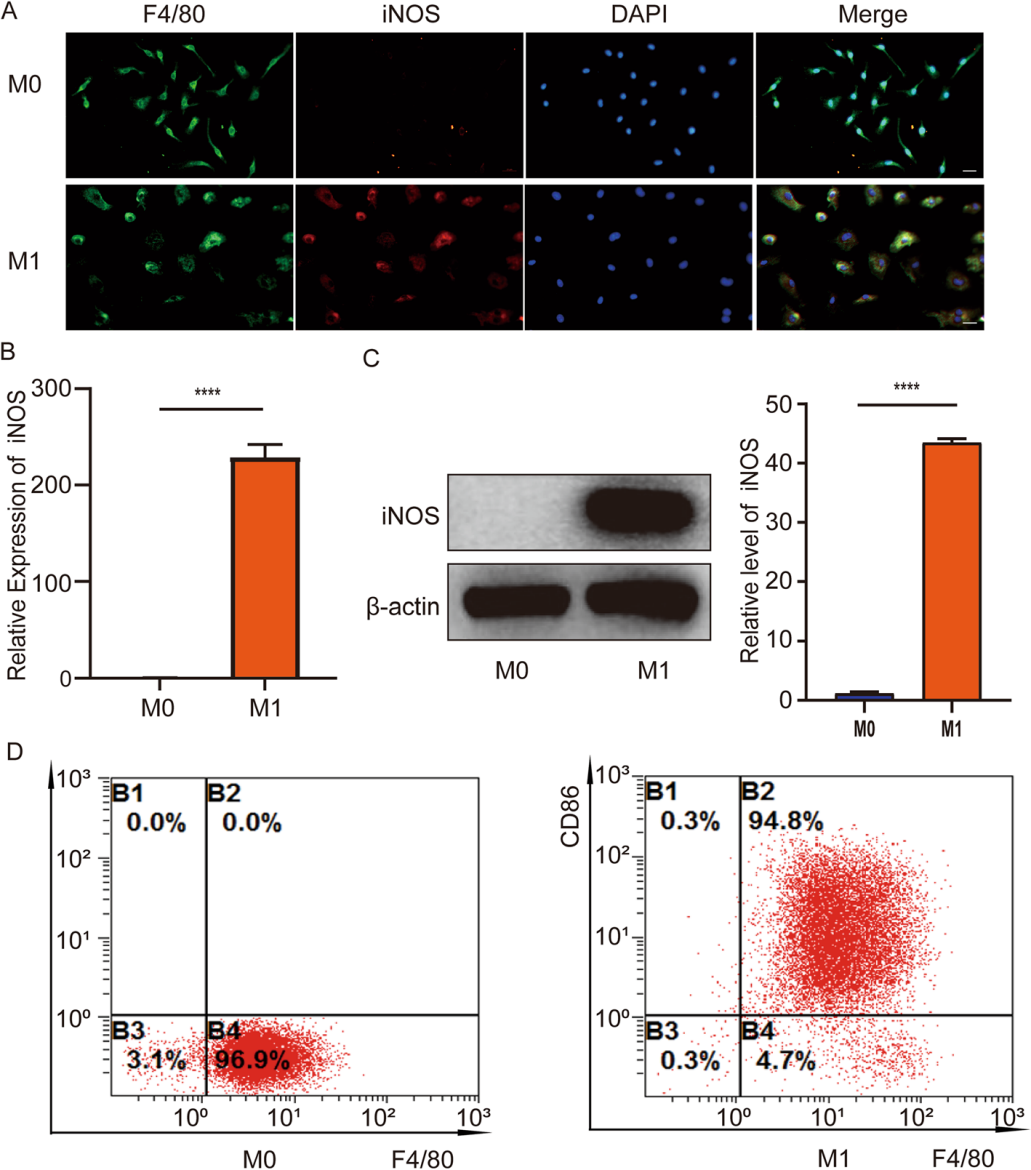
Identification and induction of macrophages. **A** Immunofluorescence staining was performed. Red shows the M1 macrophage marker iNOS. Green shows macrophage marker F4/80. Blue shows nuclear staining (DAPI). Scale bar, 50 μm. **B** RT-PCR was used to detect the expression of iNOS in M0 macrophage groups and M1 macrophage groups. **C** The protein expression level of iNOS was determined by western-blotting analysis. **D** Identification of M0 (F4/80) macrophages and M1 macrophages (F4/80, CD86) by flow cytometry. *n* = 3 per group. ****P < 0.0001

After SCI, large quantities of macrophages are recruited to the injured area, and M1 macrophages occupy the main direction and play a critical role in the damaged state [8, 26]. Hence, we extract primary macrophages from mice and induce M1 phenotype polarization in vitro. Further, identification of the primary cells was also necessary. We used the macrophage marker F4/80 and the M1 macrophage marker iNOS for immunofluorescence. The results showed that F4/80 was significantly expressed in M0 macrophages while iNOS was hardly expressed. F4/80 and iNOS were significantly expressed in M1 macrophages induced by LPS + IFN-γ (Fig. 1A). The results of RT-PCR and western blotting assay showed that the expression of iNOS in M1 macrophages was significantly higher than that in M0 macrophages (Fig. 1B, C). Flow cytometry showed that about 96.9% of the cells expressed M0 macrophage marker F4/80. About 94.8% of the cells expressed M1 macrophage markers F4/80 and CD86 (Fig. 1D). These data show that the extraction and induction of macrophages are successful.

### lncRNA TUG1 as a potential target of PBM

**Fig. 2:**
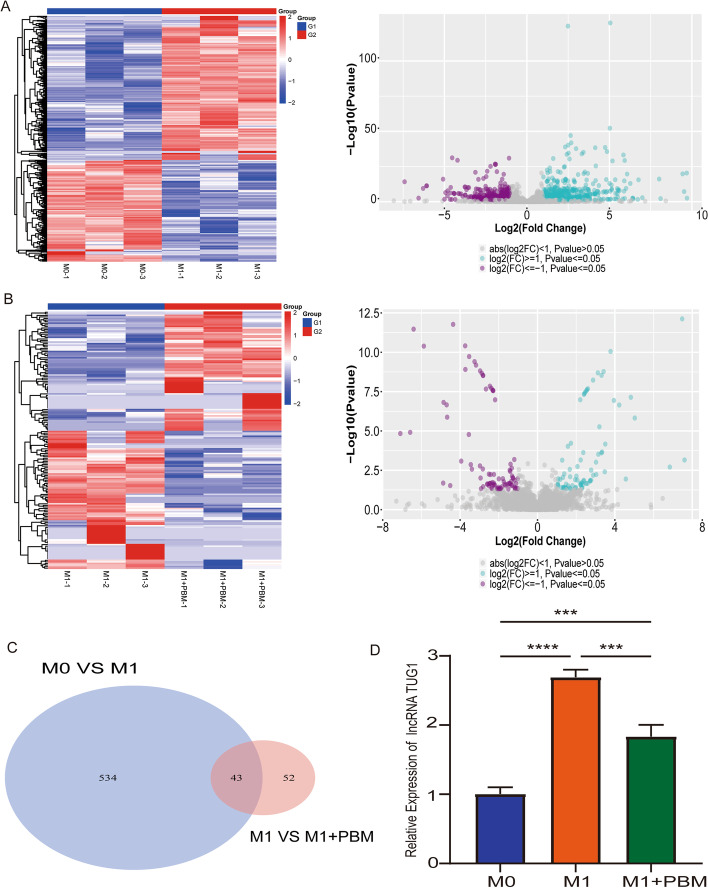
lncRNA TUG1 as a potential target of PBM. **A** Differentially expressed lncRNAs in M0 group versus M1 group were presented as heat map and volcano map. **B** Differentially expressed lncRNAs in M1 group versus M1 + PBM group were presented as heat map and volcano map. **C** Using Venn diagram to present the difference lncRNAs co-expressed between the two groups (M0 versus M1, M1 versus M1 + PBM). **D** RT-PCR was used to detect the expression of lncRNA TUG1. *n* = 3 per group. ***P < 0. 001, ****P < 0.0001

Macrophages mainly present three phenotypes: unpolarized M0 macrophages, proinflammatory M1 macrophages, and anti-inflammatory M2 macrophages [8]. In previous research, we found that PBM inhibited the polarization of M1 macrophages, but the potential target is still unclear [25]. Therefore, we sought to identify the potential target of PBM by transcriptome sequencing. We divided the cell into three groups: M0 macrophages group (M0 group), M1 macrophages group (M1 group), and M1 macrophage treated with PBM group (M1 + PBM group). Heat maps and volcano maps showed the differential genes between M0 macrophages and M1 macrophages. The gene expression profile showed that 577 differentially expressed lncRNAs were found (Fig. 2A). As shown in Fig. 2B, we revealed the differential genes in M1 macrophages group versus M1 macrophage treated with PBM group by heat map and volcano map, and 95 differentially expressed lncRNAs were found. All DElncRNAs are described in Additional file 4. Venn diagram showed that there were 43 intersection genes in M0 group, M1 group, and M1 + PBM group (Fig. 2C). We further screened the differential genes by setting fold change ≥ 2 and *P* value ≤ 0.01. There are five genes that meet the requirements: lncRNA TUG1, lncRNA miR142HG, lncRNA A230009B12Rik, lncRNA Trerf1, and lncRNA 1700113A16Rik. We detected the expression of five lncRNAs through RT-PCR, and the results indicated that only the expression of TUG1 was increased in M1 group and decreased in M1 + PBM group (Fig. 2D). The expression of the other four lncRNAs is inconsistent with the sequencing results (Additional file 1: Fig. S1A). As a “star molecule,” lncRNA TUG1 has been widely reported in promoting cancer progression, regulating inflammatory response, nerve regeneration, and osteogenic differentiation, and plays an important role in a variety of diseases [27]. Therefore, we considered that TUG1 may be the potential target of PBM and further explored its function.

### Knockdown of lncRNA TUG1 inhibits macrophage polarization and inflammation and promotes neuronal axon growth

**Fig. 3:**
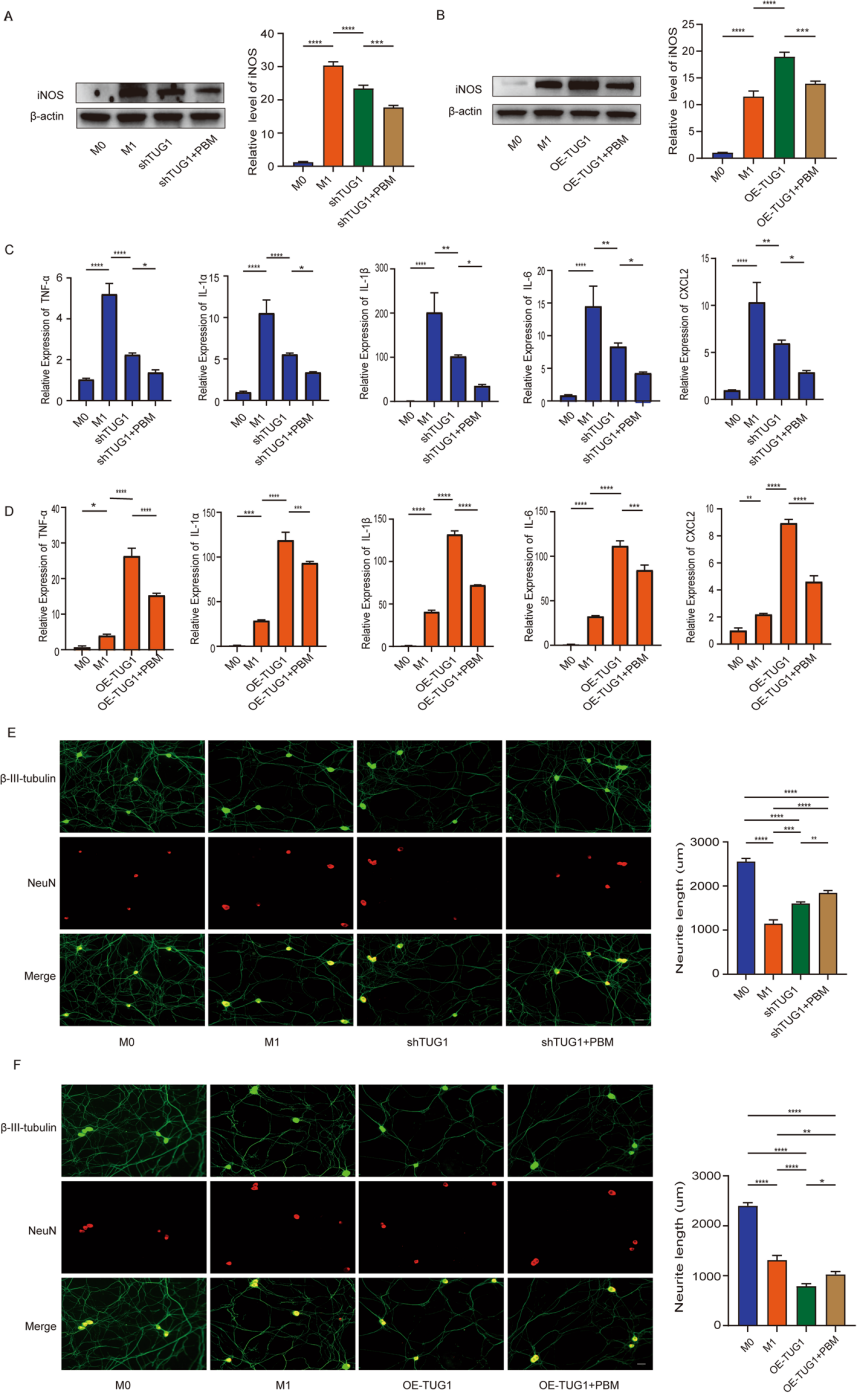
Knockdown of lncRNA TUG1 inhibits macrophage polarization and inflammation and promotes neuronal axon growth. **A** After transfection with TUG1 knockdown adenovirus, the protein expression of iNOS was detected by western blotting assay. **B** After transfection with TUG1 overexpression adenovirus, the protein expression of iNOS was detected by western blotting assay. **C** After knockdown of TUG1, RT-PCR detected the expression of TNF-α, IL-1α, IL-1β, IL-6, and CXCL2. **D** After overexpression of TUG1, RT-PCR detected the expression of TNF-α, IL-1α, IL-1β, IL-6, and CXCL2. **E**, **F** Immunofluorescence was used to observe the effect of MCM on axonal growth of DRG. Green indicates β-III-tubulin; red indicates NeuN. Scale bar, 50 μm. *n* = 3 per group. *P < 0.05, **P < 0.01, ***P < 0.001, ****P < 0.0001

To further explore the function of TUG1 in BMDMs, we constructed adenoviruses with TUG1 knockdown and overexpression and selected the best efficiency for transfection (Additional file 1: Fig. S1B-C). We constructed three TUG1 knockdown adenoviruses, and the results showed that only after transfection with shTUG1-1 adenovirus did the expression of TUG1 decrease significantly (shTUG1-1 has the best knockdown efficiency at 300 MOI). After transfection with shTUG1-2 and shTUG1-3 adenoviruses, the expression of TUG1 did not change (*P* > 0.05). Therefore, we chose shTUG1-1 with 300MOI for further experiments. Our results showed that knockdown of TUG1 (shTUG1) in M1 macrophages reduced the expression of iNOS. After knockdown of TUG1 and PBM treatment (shTUG1 + PBM), the expression of iNOS further decreased (Fig. 3A). On the contrary, overexpression of TUG1 (OE-TUG1) in M1 macrophages increased iNOS expression, while overexpression of TUG1 combined with PBM treatment (OE-TUG1 + PBM) could inhibit the increase of iNOS expression (Fig. 3B). In addition, RT-PCR results showed that the expression levels of related inflammatory cytokines and inflammatory chemokines (TNF-α, IL-1α, IL-1β, IL-6, CXCL2) was consistent with that of iNOS (Fig. 3C, D). These results suggest that TUG1 knockdown inhibits the polarization and inflammation of BMDMs and overexpression has the opposite effect. In addition, we explored the effect of M1 macrophage culture medium with knockdown and overexpression of TUG1 on DRG neurons. We found that the axon length of DRG decreased with the addition of M1 macrophage culture medium, increased in M1 macrophage culture medium with shTUG1, and further increased in M1 macrophage culture medium treated with shTUG1 + PBM treatment (Fig. 3E). Conversely, adding M1 macrophage culture medium with OE-TUG1 to DRG could aggravate the decrease of axon length, while axon growth was restored in the OE-TUG1 + PBM group (Fig. 3F). These results indicate that knockdown of TUG1 in M1 macrophages can reduce the toxicity of DRG, while overexpression of TUG1 increases the toxicity of DRG.

### Construction of lncRNA TUG1-miRNA-mRNA network

**Fig. 4:**
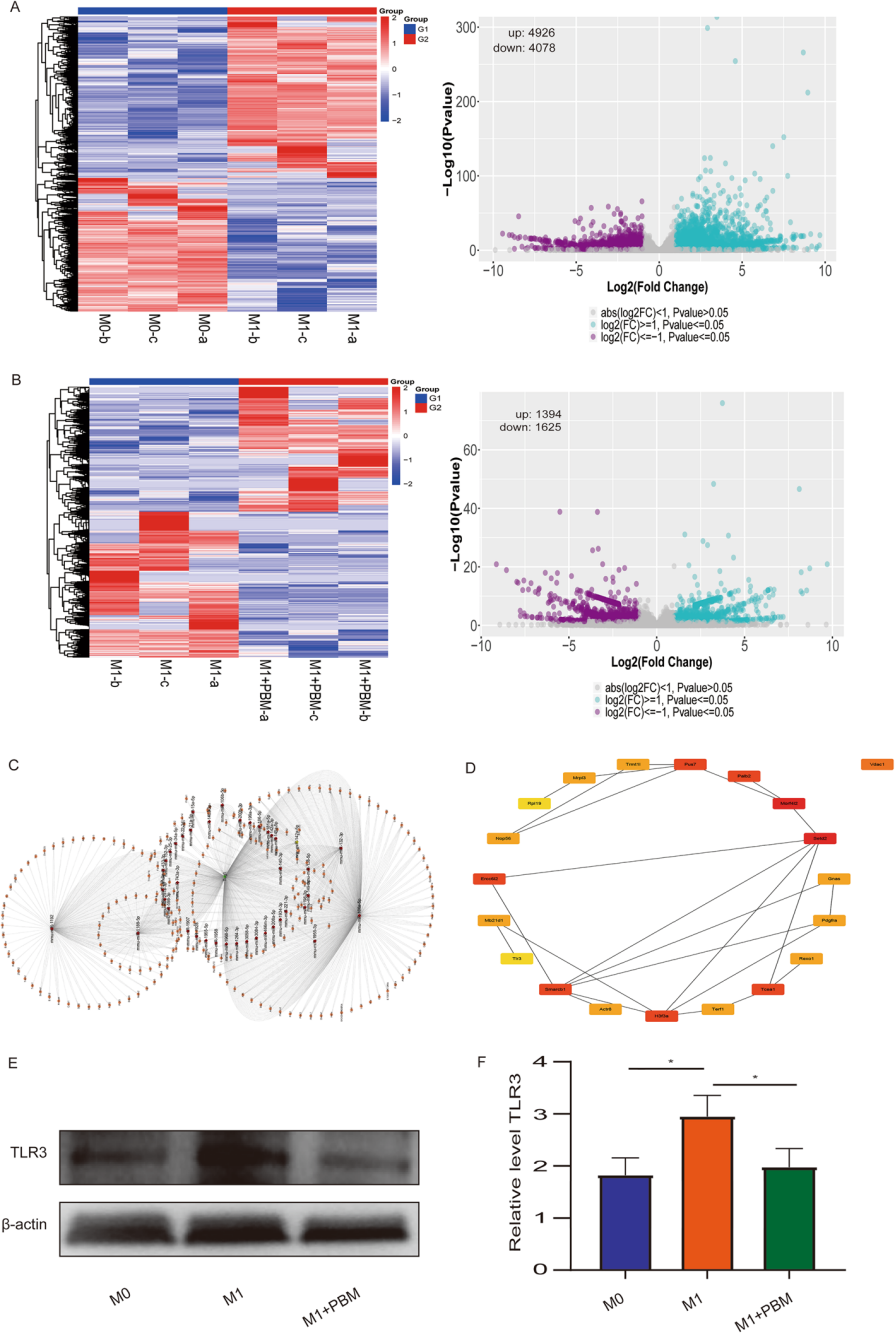
Construction of lncRNA TUG1-miRNA-mRNA network. **A** Differentially expressed mRNAs in M0 group versus M1 group were presented as heat map and volcano map. **B** Differentially expressed mRNAs in M1 group versus M1 + PBM group were presented as heat map and volcano map. **C** Bioinformatics analysis of potential TUG1-miRNA-mRNA networks with TUG1 as the core. **D** The Cytoscape software analysis hub-gene is presented (TOP20). **E** The expression of TLR3 detected by western blotting was consistent with the sequencing results. *n* = 3 per group. ****P < 0.0001

Our mRNA sequencing results showed that there were 4807 differentially expressed genes in M0 versus M1 group and 2188 differentially expressed mRNA in M1 versus M1 + PBM group (Fig. 4A, B). All DEmRNAs are described in Additional file 5. We took the intersection gene from the differential mRNAs of the two groups and constructed ceRNA network with TUG1 and miRNA (Fig. 4C). All genes in ceNRA network are presented in Additional file 6. To further screen the potential ceRNA network, we selected mRNAs (highly expressed in M1 group and lowly expressed in M1 + PBM group) and the differential mRNAs in the ceRNA network to take intersection genes, and found that 93 genes were obtained (Additional file 1: Fig. S1D). For these 93 genes, we used Cytoscape software to construct hub-gene and showed the top 20 genes (Fig. 4D). Among the 20 genes, we screened the genes with fold change ≥ 2 and *P* value ≤ 0.01 after PBM treatment. Seven genes were obtained: *Tlr3, Actr8, Terf1, Pdgfra, Palb2, Morf4l2*, and *Pus7*. It is worth noting that the Toll-like receptor signaling pathway involved by TLR3 was significantly enriched in PBM treatment group according to KEGG analysis (Additional file 1: Fig. S1E). Therefore, we selected TLR3 for verification. RT-PCR results showed that TLR3 was highly expressed in M1 macrophages and decreased after PBM treatment (Additional file 1: Fig. S1F) Similarly, TLR3 expression at the protein level was consistent with sequencing results (Fig. 4E, F). Besides, TUG1-miR-1192-TLR3 had the possibility of forming ceRNA network in bioinformatics analysis. Therefore, the TUG1-miR-1192-TLR3 axis was chosen for further research.

### TUG1 and TLR3 are direct targets of miR-1192

**Fig. 5:**
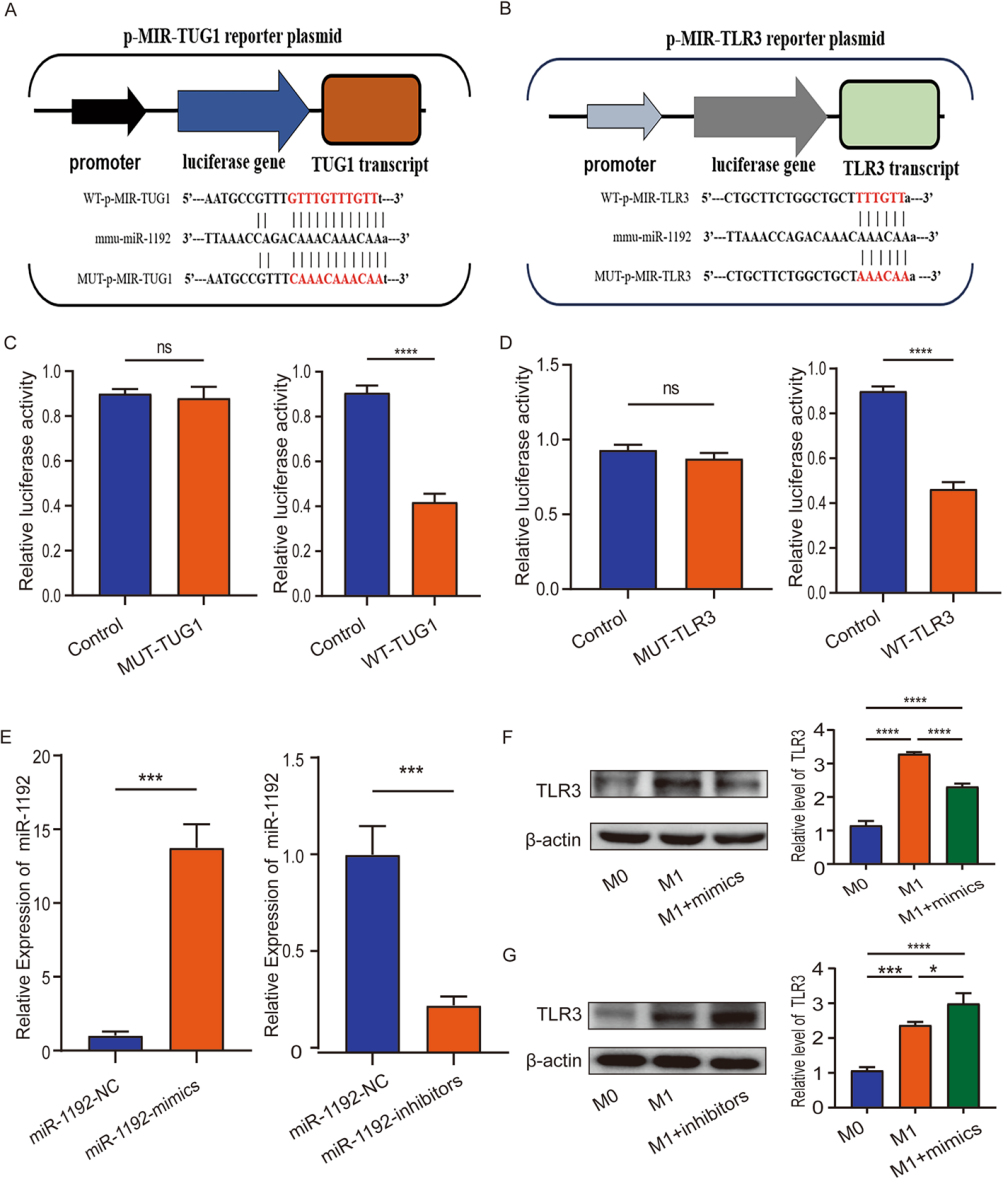
TUG1 and TLR3 are direct targets of miR-1192. **A** The binding sites of miR-1192 and TUG1 were predicted by starBase database. **B** The binding sites of miR-1192 and TLR3 were predicted by starBase database. **C**, **D** miR-1192-mimics could inhibit the luciferase activity of WT-TUG1 and WT-TLR3, but had no effect on the luciferase activity of MUT-TUG1 and MUT-TLR3. **E** RT-PCR was used to detect the transfection efficiency of miR-1192 mimics and inhibitors. **F**, **G** Western blotting was used to detect the expression level of TLR3 after transfection of miR-1192 mimics and inhibitors. *n* = 3 per group. *P < 0.05, ***P < 0.001, ****P < 0.0001. *ns* not significant

Firstly, we performed cell fraction assay to analyze the localization of TUG1 in the cytoplasm and nucleus of M1 macrophages. RT-PCR results showed that TUG1 is mainly expressed in the cytoplasm (Additional file 1: Fig. S1G). Further, we predicted the binding sites of TUG1-miR-1192 and miR-1192-TLR3 through starBase database and constructed wild-type plasmids and mutant plasmids of TUG1 and TLR3, respectively (Fig. 5A, B). Transfection in 293T cells showed that miR-1192 mimics inhibited the luciferase activity of wild-type TUG1, but had no effect on the luciferase activity of mutant TUG1 (Fig. 5C). Similarly, miR-1192 mimics increased the luciferase activity of wild-type TLR3, but had no effect on the luciferase activity of mutant TLR3 (Fig. 5D). Finally, we transfected miR-1192 mimics and inhibitors in BMDMs, and transfection efficiency was detected by RT-PCR (Fig. 5E). The results showed that miR-1192 mimics reduced TLR3 expression in M1 macrophages and miR-1192 inhibitors promote TLR3 expression (Fig. 5F, G). These results indicate that TUG1 and TLR3 are direct targets of miR-1192, and miR-1192 can bind to TLR3 and inhibit its expression.

### TUG1-miR-1192/TLR3 axis regulates macrophage polarization and inflammation

**Fig. 6:**
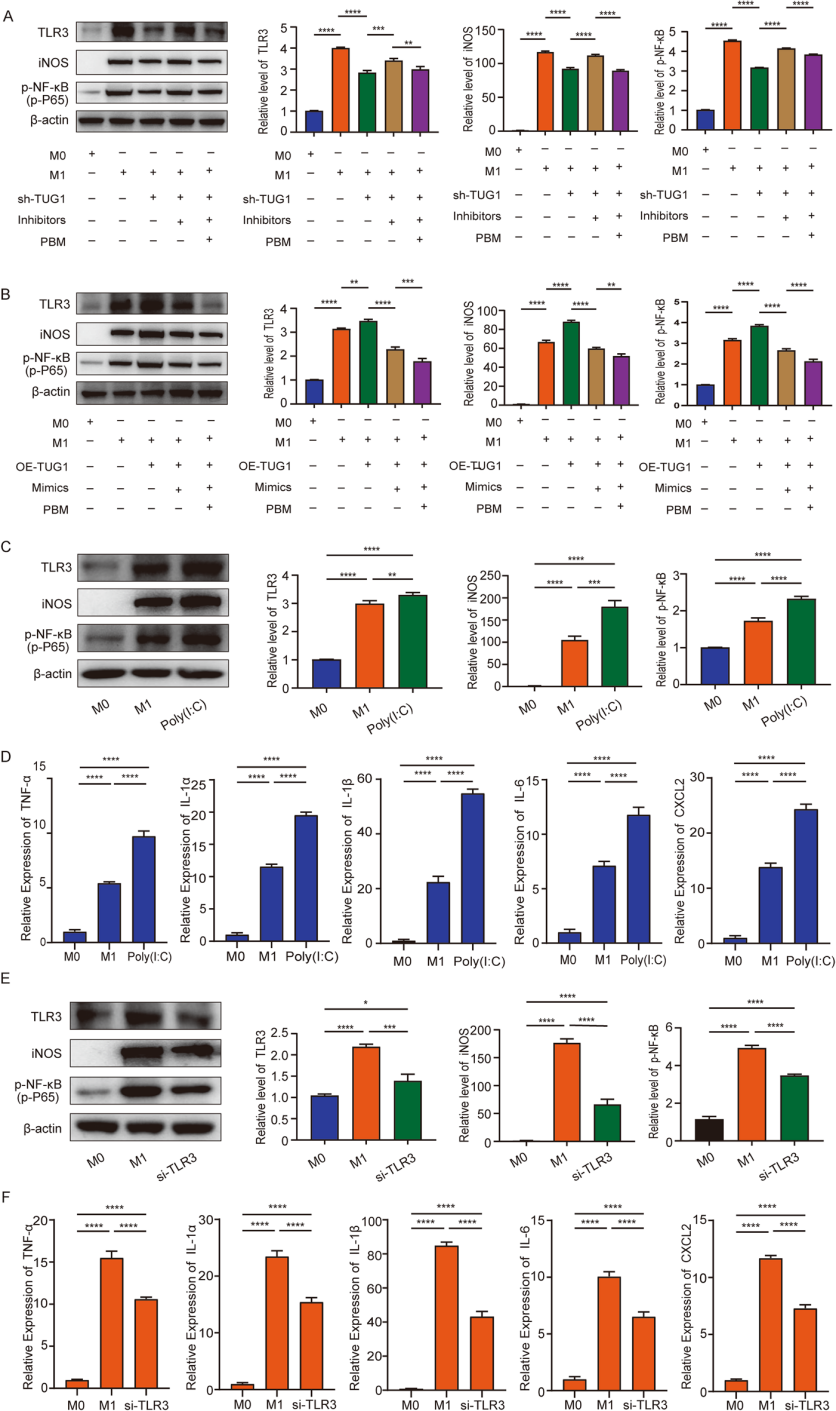
TUG1-miR-1192/TLR3 axis regulates macrophage polarization and inflammation. **A** Western blotting was used to detect the expression level of TLR3, iNOS, and p-NF-κB (p-p65), which was rescued by shTUG1 and miR-1192 inhibitor treatment. **B** Western blotting was used to detect the expression level of TLR3, iNOS, and p-NF-κB, which was rescued by OE-TUG1 and miR-1192 mimics treatment. **C**, **D** After overexpression of TLR3, western blotting was used to detect the expression of TLR3, iNOS, and p-NF-κB. RT-PCR detected the expression of TNF-α, IL-1α, IL-1β, IL-6, and CXCL2. **E**, **F** After knockdown of TLR3, western blotting was used to detect the expression of TLR3, iNOS, and p-NF-κB. RT-PCR detected the expression of TNF-α, IL-1α, IL-1β, IL-6, and CXCL2. *n* = 3 per group. *P < 0.05, **P < 0.01, ***P < 0. 001, ****P < 0.0001

To explore the specific mechanism of TUG1-miR-1192-TLR3, we carried out the “rescue experiment.” Western blotting results showed that knocking down TUG1 in M1 macrophages could inhibit the expression of TLR3. This decrease could be restored by co-transfecting shTUG1 with miR-1192 inhibitors. On this basis, PBM irradiation can inhibit the recovery of TLR3 expression (Fig. 6A). At the same time, we detected the expression level of iNOS and found that it was consistent with TLR3 (Fig. 6A). In addition, it has been reported that TLR3 can promote the activation of p-NF-κB (p-P65) and aggravate the inflammatory response, so we also detected the expression of p-NF-κB. The results showed that the protein level of p-NF-κB changed with the change of TLR3 (Fig. 6A). On the other hand, overexpression of TUG1 in M1 macrophages promoted TLR3 expression. The expression of TLR3 was inhibited by co-transfecting OE-TUG1 with miR-1192 mimics and PBM irradiation further suppressed the expression of TLR3 (Fig. 6B). The expression of iNOS and p-NF-κB was also consistent with TLR3 (Fig. 6B). These data suggest that TUG1 can regulate the expression of TLR3 by sponging miR-1192, and the expression of TLR3 promotes the activation of p-NF-κB signaling pathway. Furthermore, we explored the effect of TLR3 for iNOS expression. We added TLR3 expression agonists (Poly(I:C)) to M1 macrophages and detected the expression of TLR3 by RT-PCR (Additional file 1: Fig. S1H). The results showed that overexpression of TLR3 could promote the expression of iNOS and p-NF-κB, and the expression of TNF-α, IL-1α, IL-1β, IL-6, and CXCL2 was also increased (Fig. 6C, D). Next, we synthesized the siRNA of TLR3 and transfected it into M1 macrophages, and RT-PCR showed that siTLR3-1 was the most efficient (Additional file 1: Fig. S1I). The results showed that transfection of siTLR3-1 in M1 macrophages inhibited the expression of iNOS and p-NF-κB, and the expression of inflammatory related molecules also decreased (Fig. 6E, F). This result is consistent with our conjecture, indicating that PBM can regulate macrophage polarization and inflammatory response through the TUG1-miR-1192/TLR3 axis.

### PBM inhibits the expression of TUG1, TLR3, and inflammatory cytokines in SCI mice

**Fig. 7:**
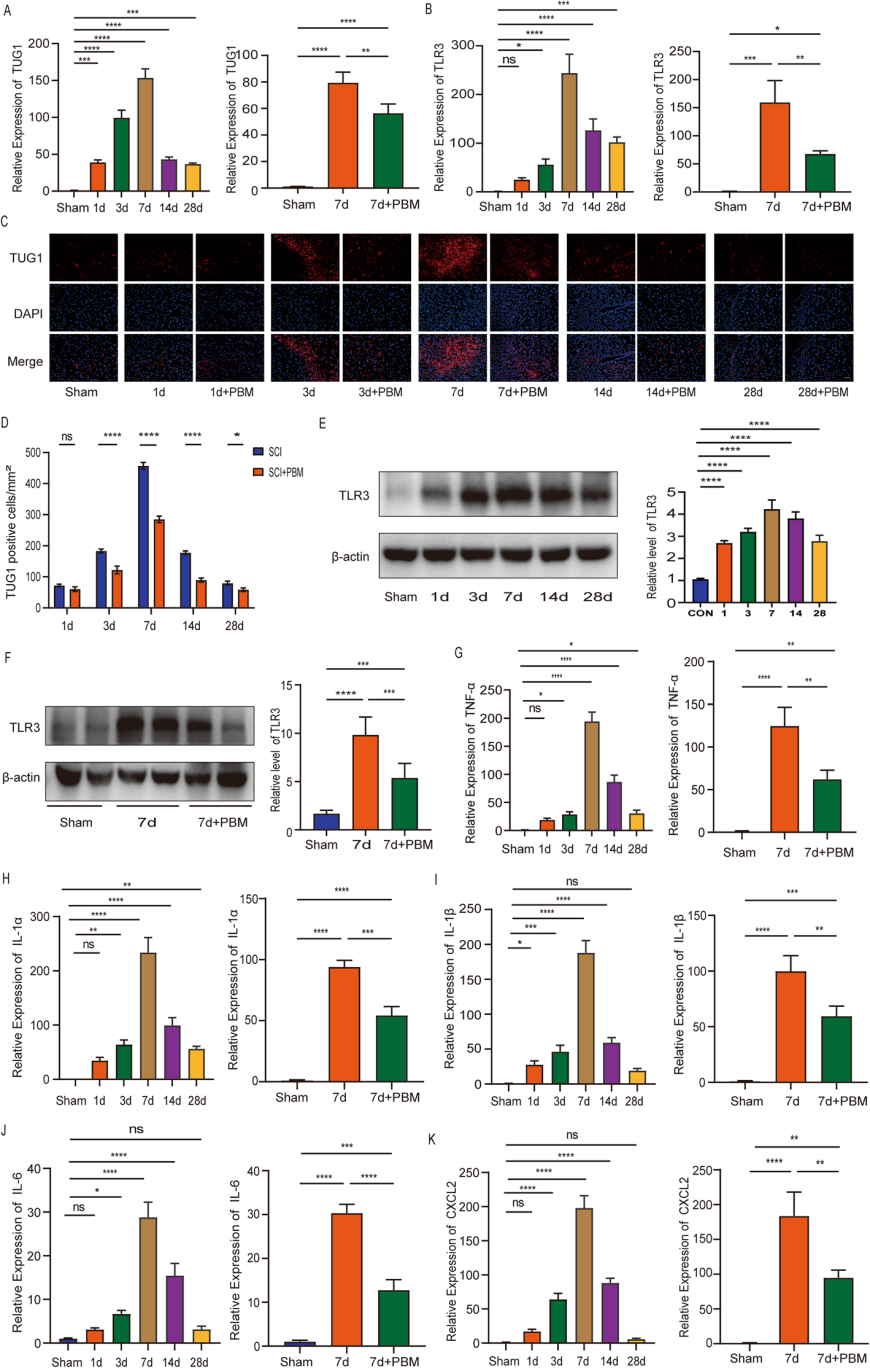
PBM can inhibit the expression of TUG1, TLR3, and inflammatory cytokines in SCI mice. **A**, **B** RT-PCR was used to detect the expression of TUG1 and TLR3 at 1, 3, 7, 14, and 28 days after SCI. In addition, RT-PCR results showed that the expression of TUG1 and TLR3 decreased after 7 days of PBM treatment. **C**, **D** FISH assay was used to detect the expression of TUG1 in SCI group and PBM group at 1, 3, 7, 14, and 28 days after SCI. Scale bar, 200 μm. **E** The protein expression level of TLR3 was detected by western blotting at 1, 3, 7, 14, and 28 days after SCI. **F** PBM treatment reduces TLR3 expression. (**G**–**K**) RT-PCR was used to detect the expression of TNF-α, IL-1α, IL-1β, IL-6, and CXCL2 at 1, 3, 7, 14, and 28 days after SCI, and the expression of TNF-α, IL-1α, IL-1β, IL-6, and CXCL was reduced after 7 days of PBM treatment. *n* = 3 per group. *P < 0.05, ***P < 0.001, ***P < 0.001, ****P < 0.0001. *ns* not significant

First, we detected the expression of TUG1 and TLR3 in normal mice and 1, 3, 7, 14, and 28 days after SCI by RT-PCR. It was found that the expression of TUG1 and TLR3 was the highest at 7 days after SCI, while the expression of TUG1 and TLR3 decreased after 7 days of PBM therapy (Fig. 7A, B). In addition, we performed the FISH assay at 1, 3, 7, 14, and 28 days SCI. The results showed that, compared with SCI group, PBM therapy group could effectively reduce the expression of TUG1 at 3, 7, 14, and 28 days after the injury (Fig. 7C, D). Through western blotting assays, we found that the expression of TLR3 was consistent with the result of RT-PCR, which was the highest at 7 days after SCI and decreased after 7 days of PBM therapy (Fig. 7E, F). Furthermore, we detected the expression of TNF-α, IL-1α, IL-1β, and IL-6 and CXCL2 at 1, 3, 7, 14, and 28 days SCI. The results showed that expression peaked at 7 days after injury and reduced after PBM therapy (Fig. 7G–K).

### PBM promotes neuronal survival and motor function recovery after SCI

**Fig. 8:**
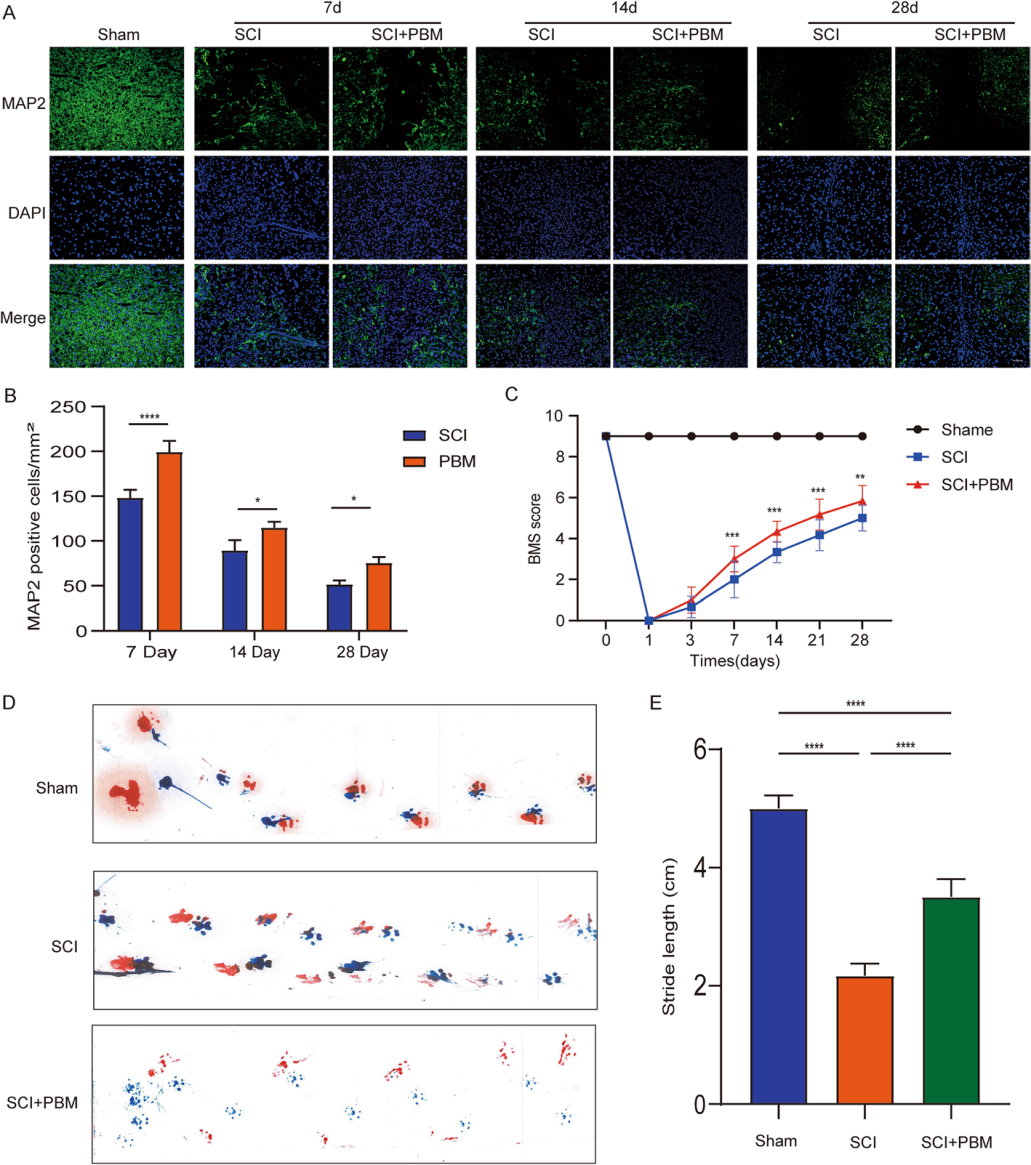
PBM promotes neuronal survival and motor function recovery after SCI. **A**, **B** Immunofluorescence was used to stain neurons at 7, 14, and 28 days after SCI. Compared with the injured group, PBM can promote the survival of neurons (*n* = 3 per group). Scale bar, 200 μm. **C** The BMS score was used to evaluate the recovery of motor function in mice at 1, 3, 7, 14, and 28 days after SCI (*n* = 6 per group). **D**, **E** At 28 days, the footprints of each group were obtained by gait analysis, The average step length is counted (*n* = 6 per group). *P < 0.05, ***P < 0.001, ***P < 0.001, ****P < 0.0001

**Fig. 9:**
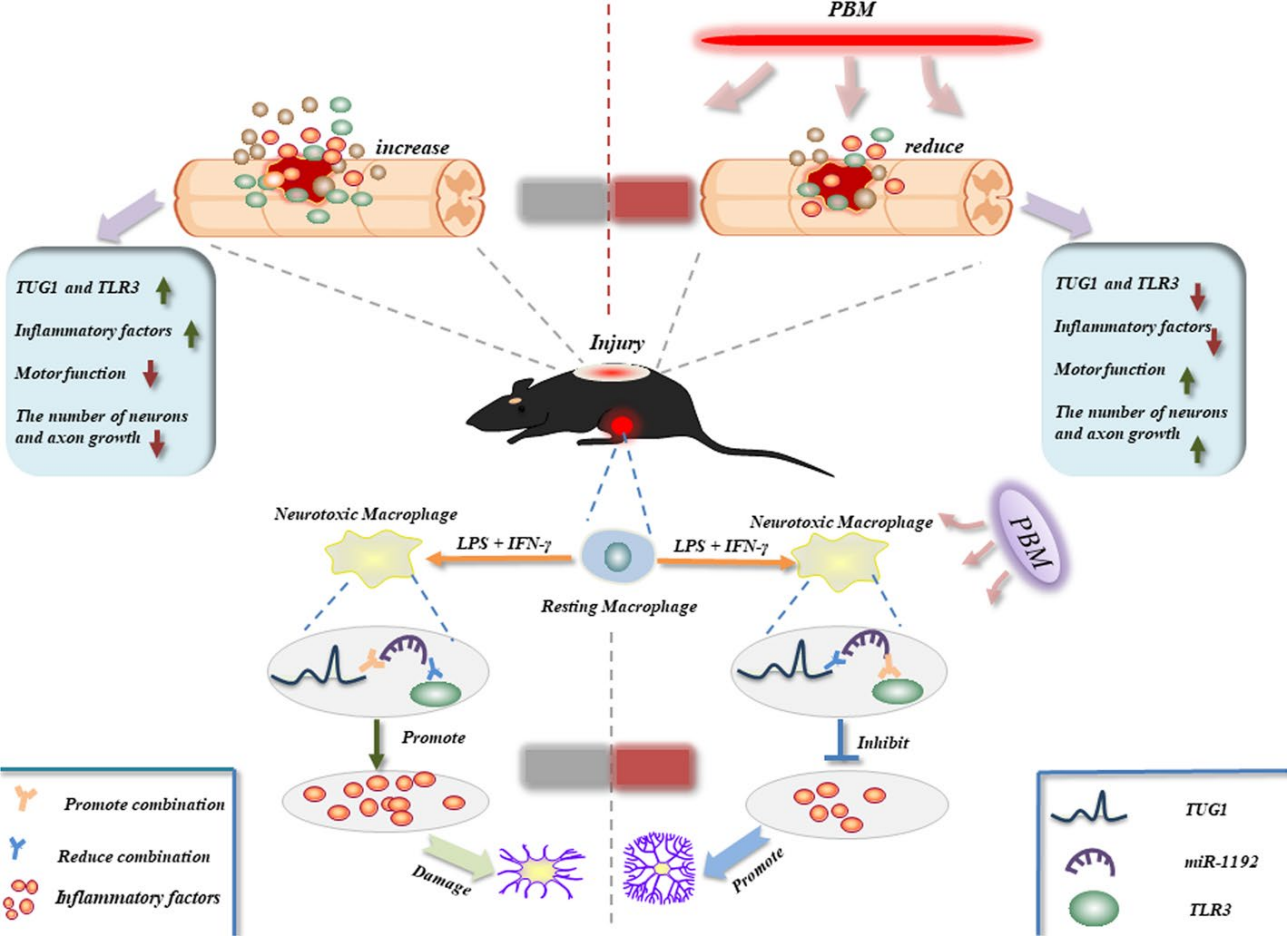
Schematic diagram showing the potential mechanism of PBM to promote SCI repair. At the cellular level, the expression of lncRNA TUG1 increased after the induction of M1 macrophages, which promoted the polarization and inflammatory response of M1 macrophages and aggravated neuronal damage. In mechanism, tug1 can promote TLR3 expression by adsorbing miR-1192, while PBM inhibits this pathway. PBM reduced the expression of TUG1, TLR3 and inflammatory cytokines in spinal cord tissue, and promoted the recovery of motor function

Previous studies have reported that reducing the expression of inflammatory cytokines after SCI helps to alleviate the injury and stimulation of neurons [28]. Our research has shown that PBM can reduce the inflammatory response after SCI, so can it promote the survival of neurons. We performed MAP2 (the marker of neuron) staining on SCI spinal cord tissue at 7, 14, and 28 days after injury. The results showed that PBM could effectively promote the survival of neurons compared with the injury group (Fig. 8A, B). In addition, after treatment with PBM for 28 days after injury, BMS score showed that PBM can effectively promote the functional recovery of mice from 7 days after SCI (Fig. 8C). At the same time, gait analysis showed that PBM could increase the step length of mice compared with SCI group at 28 days (Fig. 8D, E). Finally, we drew a diagram of the mechanism of PBM inhibiting macrophage polarization and promoting SCI repair (Fig. 9).

## Discussion

Many studies support that lncRNA, as an important molecule, plays an important role in SCI progression [29]. Aberrantly expressed lncRNAs can regulate cell polarization, inflammatory reaction, and nerve cell survival in the injured area [30–33]. In this study, our transcriptome sequencing showed that the lncRNA and mRNA profiles in BMDMs irradiated with PBM were significantly changed. Further studies found that high levels of TUG1 can promote the polarization and inflammatory response of M1 macrophages and reduce the axonal growth of neurons, and lncTUG1-miRNA-mRNA network plays a key role in PBM treatment of SCI. To the best of our knowledge, this is the first report to identify TUG1 as a potential target for PBM and clarify the mechanism of TUG1 in SCI. lncRNA TUG1 was initially detected in taurine-treated mouse retinal cells, and upregulation of TUG1 is critical for retinal development [34]. Recent studies show that TUG1 is closely related to human disease progression and could regulate cancer progression, inflammatory reaction and nerve regeneration [35–37]. It has been reported that TUG1-mediated ceRNA can promote neural apoptosis and thus aggravate SCI progression in the rat model. This study emphasizes that the lncRNA TUG1-miR-338/bik axis is a potential mechanism to reduce apoptosis of PC-12 cells [32]. In our study, we screened TUG1 as a potential target of PBM by transcriptome sequencing. Further studies mainly elucidated the regulation of TUG1 on M1 macrophage polarization and inflammatory response as well as its effect on axon growth. We found that knockdown of TUG1 inhibits M1 macrophage polarization and inflammatory response, while overexpression of TUG1 promotes the polarization of M1 macrophages and thus the inflammatory response. These results indicate that lncRNA TUG1 is the target of PBM therapy that regulates the polarization of macrophages and inflammation. In addition, DRG cultured with MCM has improved axonal growth. We consider that this may occur because TUG1 regulates the expression of inflammatory cytokines releases these cytokines to aggravate or alleviate neuronal damage, or TUG1 acts directly on neurons through intercellular transmission of exosomes. The ceRNA hypothesis is the most common and classical behavior of lncRNA [38]. This suggests that RNA transcripts with the same miRNA response element (MRE) can competitively bind to miRNA and act as RNA sponges to prevent miRNA from binding to its target site [39, 40]. Existing studies have shown that imbalanced expression of ceRNA network can change cell proliferation, metastasis, abnormal differentiation, and apoptosis [41]. To further explore the mechanism of TUG1, we used bioinformatics analysis to identify the TUG1-miR-1192-TLR3 network. In this network, TUG1 can compete with TLR3 to bind miR-1192, thereby reducing the inhibitory effect of miR-1192 on TLR3. This pathway may be the key reason for the function of PBM. In addition, in vivo experiments, TUG1 reached its peak at 7 days after injury and then began to decrease, while PBM treatment after injury could inhibit the expression of TUG1. Our results highlight the involvement of TUG1 in PBM treatment in vivo and in vitro.

miRNA is a noncoding RNA with a length of about 20–22 nucleotides [42, 43]. Recently, considerable attention has been focused on the sponge effect of miRNAs. miRNAs regulate gene expression by directly binding mRNA and subsequently inhibiting mRNA translation or inducing mRNA degradation [44]. Previous studies have shown that miR-1192 can enhance Runx2-induced osteogenic differentiation through targeted inhibition of HB-EGF expression, and the upregulation of miR-1192 can play a cardioprotective role and inhibit inflammatory response [45, 46]. TLR3 can act as an MRE for miR-1192 by bioinformatics analysis. Previous studies have shown that the elevation of TLR3 can promote the release of inflammatory cytokines and the activation of downstream NF-κB signaling pathway [47]. In addition, TLR3 can be degraded by binding with miRNA [48]. Here, we analyzed whether miR-1192 could inhibit the expression of TLR3. The results showed that mimics of miR-1192 inhibited TLR3 expression, while inhibitors of miR-1192 increased TLR3 expression. In addition, TUG1 regulates the expression of TLR3, which can be affected by miR-1192. Therefore, TLR3 appears to be a downstream target of the ceRNA network. We further knocked down and overexpressed TLR3, and observed that the expression of iNOS, inflammatory cytokines, and NF-κB signaling pathway is consistent with the regulation of TUG1. In vivo, TLR3 expression was consistent with TUG1 and inflammatory cytokine expression. Inflammatory response is an important process of SCI. Massive release of inflammatory cytokines has a significant impact on the deterioration of SCI. In our study, it was shown that inflammatory cytokines reached a peak at 7 days after SCI, which is consistent with previous reports. In vivo, PBM treatment significantly inhibits the release of inflammatory cytokines.

However, there are also some limitations. In this study, we did not verify the role of the TUG1/miR-1192/TLR3 axis in regulating SCI at the animal level. More importantly, physiological differences between lower animals and humans need to be taken into account, which may lead to parameter differences between animals and humans required for PBM treatment. These are the important directions of our next research.

## Conclusions

Our study identified the expression profile of differential genes in PBM inhibition of macrophage polarization by transcriptome sequencing and bioinformatics analysis. We clarified that lncRNA TUG1 is a target gene for PBM to regulate macrophage polarization and inflammation. PBM may regulate macrophage polarization through the lncRNA TUG1-miR-1192-TLR3 pathway, interfere with secondary inflammatory response, and promote the functional recovery of spinal cord injury. This study elucidates the possible mechanism of PBM, providing theoretical support for its use in clinical treatment of SCI.

## Supplementary Information

**Additional file 1: Fig. S1.** (**A**) The expression level of miR142HG, A230009B12Rik, Trerf1, and 1700113A16Rik were detected by RT-PCR. (**B**) The knockdown efficiency of shTUG1-1 is the highest at 300 MOI. (**C**) TUG1 overexpression was most efficient at 500 MOI. (**D**) The intersection genes are shown in the Venn diagram. (**E**) GO analysis showed that Toll-like receptor signaling pathways were enriched. (**F**) The expression level of TLR3 was detected by RT-PCR. (**G**) The localization of TUG1 in the cytoplasm and nucleus of M1 macrophages. (**H**) The overexpression efficiency of TLR3 was detected by RT-PCR. (**I**) RT-PCR showed that the knockdown efficiency of siTLR3-1 was the best.

**Additional file 2.** The specific parameters of PBM.

**Additional file 3.** Primer sequences used for Quantitative real-time PCR.

**Additional file 4.** All DElncRNAs in M0 group, M1 group and M1+PBM group.

**Additional file 5.** All DEmRNAs in M0 group, M1 group and M1+PBM group.

**Additional file 6.** All genes in ceRNA network.

## Data Availability

The datasets during and/or analyzed during the current study are available from the corresponding author on reasonable request. Transcriptome sequencing data were uploaded to the NCBI database (login number PRJNA780778).

## Abbreviations

SCI: Spinal cord injury
BMDMs: Bone-marrow-derived macrophages
PBM: Photobiomodulation
lncRNA: Long noncoding RNA
ceRNA: Competing endogenous RNA
TUG1: Taurine upregulated gene 1
UTR: Untranslated region
MCSF: Macrophage colony-stimulating factor
DRG: Dorsal root ganglion
MOI: Multiplicity of infection
qPCR: Quantitative real-time PCR
MCM: Macrophage-conditioned medium
MRE: miRNA response element
